# Retraction: Role of Hepatitis C Virus Induced Osteopontin in Epithelial to Mesenchymal Transition, Migration and Invasion of Hepatocytes

**DOI:** 10.1371/journal.pone.0216026

**Published:** 2019-04-30

**Authors:** The PLOS ONE Editors

Following publication of [1], concerns were noted in the following figures:

- Fig 1B, the HCV NS5A panel lanes 4–7 appear similar to Fig 3C panel HCV core lanes 2–5 in a 2010 *Journal of General Virology* article [2], Fig 1A ASC panel in a 2016 *Journal of Biological Chemistry* article [5] and Fig 7B Albumin panel in a *PLOS ONE* article by the same authors [6]. The 2016 *Journal of Biological Chemistry* article has been withdrawn [7] and the *PLOS ONE* article has been retracted [8].
- In discussing this matter, the authors notified the journal that Fig 2A, Huh7.5+NS5A panel is incorrect. This panel appears similar to Fig 7E Huh7.5+NS5A in a *2013 Journal of Biological Chemistry* article [3] by the same authors which has been withdrawn [4].

The authors have provided image data for Fig 1B as well as replacement images for Figs 1B and 2A which represent replication experiments. However, these data did not resolve the potential image reuse across publications.

The original image underlying published Fig 2A is unavailable, however all other primary data underlying the figures are available from the authors. Given the unresolved concerns surrounding Figs 1B and 2A as well as repeated re-use of these images in multiple publications that call into question the validity of the study findings, the *PLOS ONE* editors retract this article.

GW did not agree with retraction. JI, SM, TM, KB, and MSD did not respond.

Owing to the concerns about similarities with previously published content [2–5, 7] that is not licensed for reproduction and distribution under the terms of the Creative Commons Attribution License, Fig 1B HCV NS5A panel, Fig 2A Huh7.5+NS5A panel have been removed from the retracted article [1].
